# The Optical Nature of Myopic Changes in Retinal Vessel Caliber

**DOI:** 10.1016/j.xops.2024.100631

**Published:** 2024-10-10

**Authors:** Fabian Yii, Niall Strang, Colin Moulson, Baljean Dhillon, Miguel O. Bernabeu, Tom MacGillivray

**Affiliations:** 1 Robert O Curle Ophthalmology Suite, Institute for Regeneration and Repair, The University of Edinburgh, Edinburgh, UK; 2 Centre for Clinical Brain Sciences, The University of Edinburgh, Edinburgh, UK; 3 Department of Vision Sciences, Glasgow Caledonian University, Glasgow, UK; 4 Princess Alexandra Eye Pavilion, NHS Lothian, Edinburgh, UK; 5 Centre for Medical Informatics, Usher Institute, The University of Edinburgh, Edinburgh, UK

**Keywords:** Retinal vessel caliber, Ocular magnification, Myopia, Axial length, Telecentricity

## Abstract

**Purpose:**

Dimensional measures of retinal features are subject to the optical influence of ocular magnification. We examined the impact of ocular magnification on the association between axial length (AL) and measurements of retinal vessel caliber in fundus photographs.

**Design:**

Cross-sectional study.

**Participants:**

Eighty-two normal right eyes from healthy participants aged 16 to 31 years.

**Methods:**

Central retinal arteriolar and venular equivalents (CRAE and CRVE) were derived from color fundus photographs using semiautomated software. Ordinary least squares linear regression was used to assess the influence of AL (independent variable) on CRAE and CRVE, controlling for age, sex, and ethnicity, both before and after magnification correction using different formulae. These formulae estimate magnification based on different ocular parameters: AL only (Bennnett’s formula), refractive error only (Bengtsson’s formula), and refractive error combined with keratometry (Littmann’s formula). Previous research has primarily relied on Bengtsson’s formula, which is less accurate than Bennett’s formula. We also examined the impact of treating the nontelecentric fundus camera used in this study as telecentric when applying these magnification correction formulae.

**Main Outcome Measures:**

Central retinal arteriolar and venular equivalents (in pixels).

**Results:**

Before magnification correction, increasing AL was associated with decreasing CRAE (β: −0.49, 95% confidence intervals: −0.89 to −0.09, *P* = 0.02) and CRVE (β: −0.91, 95% confidence intervals: −1.62 to −0.20, *P* = 0.01). After magnification correction, this observation was no longer evident, regardless of the correction formula applied. When inappropriately assuming the fundus camera to be telecentric, we observed a bias toward increasing magnification-corrected CRAE and CRVE with increasing AL (β coefficients were positive or became more positive), reaching statistical significance (*P* < 0.05) for CRAE corrected using Bennett’s or Littmann’s formula, and for CRVE corrected using Bennett’s formula.

**Conclusions:**

Failing to correct for ocular magnification results in apparent narrowing of vessels in longer eyes, while inappropriate assumptions about telecentricity during magnification correction introduce an optical artifact that causes apparent widening of vessels. These findings suggest that myopic changes in retinal vessel caliber are optical (not biological) in nature. Proper correction of this effect to accurately derive dimensional measures is a crucial—yet often overlooked—methodological consideration in “oculomics” research investigating retinal biomarkers of systemic conditions.

**Financial Disclosure(s):**

Proprietary or commercial disclosure may be found in the Footnotes and Disclosures at the end of this article.

Retinal alterations in myopia are generally attributed to the stretching of ocular tissues during axial elongation.^1^ Our recent work, analyzing >20 000 healthy adults in the UK Biobank, further demonstrated that changes in a wide range of retinal landmarks occurred in a highly nonlinear fashion across refractive error.^2^ Specifically, the *magnitude* of retinal changes induced by *each diopter (D) increase* in refractive error *increased* as myopia became more severe, tying in with observations that the odds of myopia-related complications increase exponentially (or quasi-exponentially), not linearly, with higher myopia.^3^ One intriguing observation from this study was that the initially positive association between spherical equivalent refraction (SER) and retinal vessel caliber, as captured by the measurements of central retinal arteriolar or venular equivalent (CRAE or CRVE), was no longer evident but changed direction after the optical influence of ocular magnification was accounted for. However, one limitation of the study was the lack of axial length (AL) information, which is a more direct measure of ocular dimensions.

Equally intriguing is the heterogeneity in results across existing studies: some suggested arteriolar/venular narrowing in myopia,^4^, ^5^, ^6^, ^7^, ^8^ while others found little evidence of an association.^9^^,^^10^ It is important to note that ocular magnification was not always accounted for in studies reporting a reduction in vessel caliber, either because this was part of the study design to investigate variations in ocular magnification induced by refractive error^4^ or because magnification correction was assumed to be unimportant,^5^^,^^6^ so the results might merely reflect the optical effect of *decreasing* magnification in longer eyes. Indeed, following magnification correction, 2 studies noted the disappearance of arteriolar and venular narrowing,^9^^,^^10^ although 2 other studies still found evidence of a reduction in CRAE and CRVE with increasing AL.^7^^,^^8^ It is also worth noting that while our previous work^2^ used Littmann’s formula^11^ for magnification correction, other equivalent studies^7^, ^8^, ^9^, ^10^ applied Bengtsson’s formula.^12^ This raises the possibility that the choice of formula might also influence the observed association between myopia and vessel caliber, as existing formulas yield varying degrees of accuracy due to the different assumptions made about the eye.^13^ Despite these uncertainties, a decrease in vessel caliber is not uncommonly cited as one line of evidence supporting the notion that a reduction in ocular blood flow might explain why high myopia protects against severe diabetic retinopathy,^6^^,^^14^, ^15^, ^16^ with the tacit but inaccurate assumption being that the evidence base supporting a biological reduction in vessel caliber in myopia is well established.

Against this backdrop, the primary aim of the present study was to investigate if the optical influence of ocular magnification might explain the arteriolar/venular narrowing in myopia reported by previous studies.^4^, ^5^, ^6^, ^7^, ^8^ We did this by comparing the association of AL with retinal vessel caliber before and after magnification correction—using Bennett’s formula,^17^ which was previously found to be the most accurate formula,^13^ Bengtsson’s formula,^12^ which was favored by previous research cited above, and Littmann’s formula,^11^ which was used in our recent work.^2^

## Methods

### Participants

A dataset comprising 96 healthy young students from the department of Vision Sciences at Glasgow Caledonian University was analyzed. The research adhered to the tenets of the Declaration of Helsinki. Following ethical approval by the School of Health and Life Sciences ethics committee (HLS/LS/A23/013) and informed consent from each participant, data from the right eyes of these participants were collected. After removing 3 eyes with signs of pathologic myopia (diffuse chorioretinal atrophy), 6 eyes with self-reported strabismus, 4 eyes with self-reported amblyopia, and 1 eye with self-reported keratoconus, 82 eyes were included in this study. Visual acuity can be assumed to be normal in these young participants on account of the absence of any self-reported ocular conditions and pathological findings based on fundus photography.

### Data Collection

Macula-centered fundus photographs with a 45-degree field of view were acquired using a DRI OCT Triton Plus (Topcon Corporation) device, while AL was measured using an optical biometer (IOL Master 500, Zeiss Medical). Refractive error and keratometry were determined using a Tonoref II autorefractometer (Nidek) under cycloplegia (1% cyclopentolate). Spherical equivalent refraction was defined as spherical power + 0.5 × cylindrical power, while corneal radius of curvature (CR) was given by the mean radius of curvature of the steepest and flattest corneal meridians.

Retinal vessel caliber was measured semi-automatically using Vessel Assessment and Measurement Platform for Images of the Retina (VAMPIRE, Universities of Edinburgh and Dundee, version 3.2), which has been detailed previously.^18^^,^^19^ Briefly, the software automatically segmented the retinal vasculature and classified each vessel as either artery or vein. The user could then manually check and correct any errors in vessel segmentation or classification if necessary. Following this, the software derived CRAE and CRVE (Knudtson’s formula)^20^ from within a region defined by an annulus measuring 0.5 to 1 optic disc diameter from the disc margin based on the widely adopted standardization introduced by the Atherosclerosis Risk in Communities study.^21^^,^^22^ The disc margin was detected automatically and corrected manually as appropriate to ensure it corresponded to the inner scleral ring and excluded peripapillary atrophy (if present). A single operator (F.Y.) performed the measurements twice—with an interval of ≥1 month between measurements (intraclass correlation coefficient: 0.77 for CRAE and 0.85 for CRVE)—and the mean values were used in all subsequent analyses. The operator was masked to participant characteristics, including AL and sex. Central retinal arteriolar and venular equivalent were expressed in pixels. Conversion to a physical unit, such as microns, was unnecessary for studying the relationship between AL and vessel caliber because all images were captured using the same fundus camera (i.e., conversion factor was constant across all images).

### Ocular Magnification Correction

Given the true size, *t*, of a retinal feature of interest, its measured or observed size, *s*, is influenced by a factor peculiar to the fundus camera, *p*, and another factor governed by the optical dimensions of the eye being imaged, *q*:^11^^,^^13^

[1] t=p×q×s

**Figure 1:**
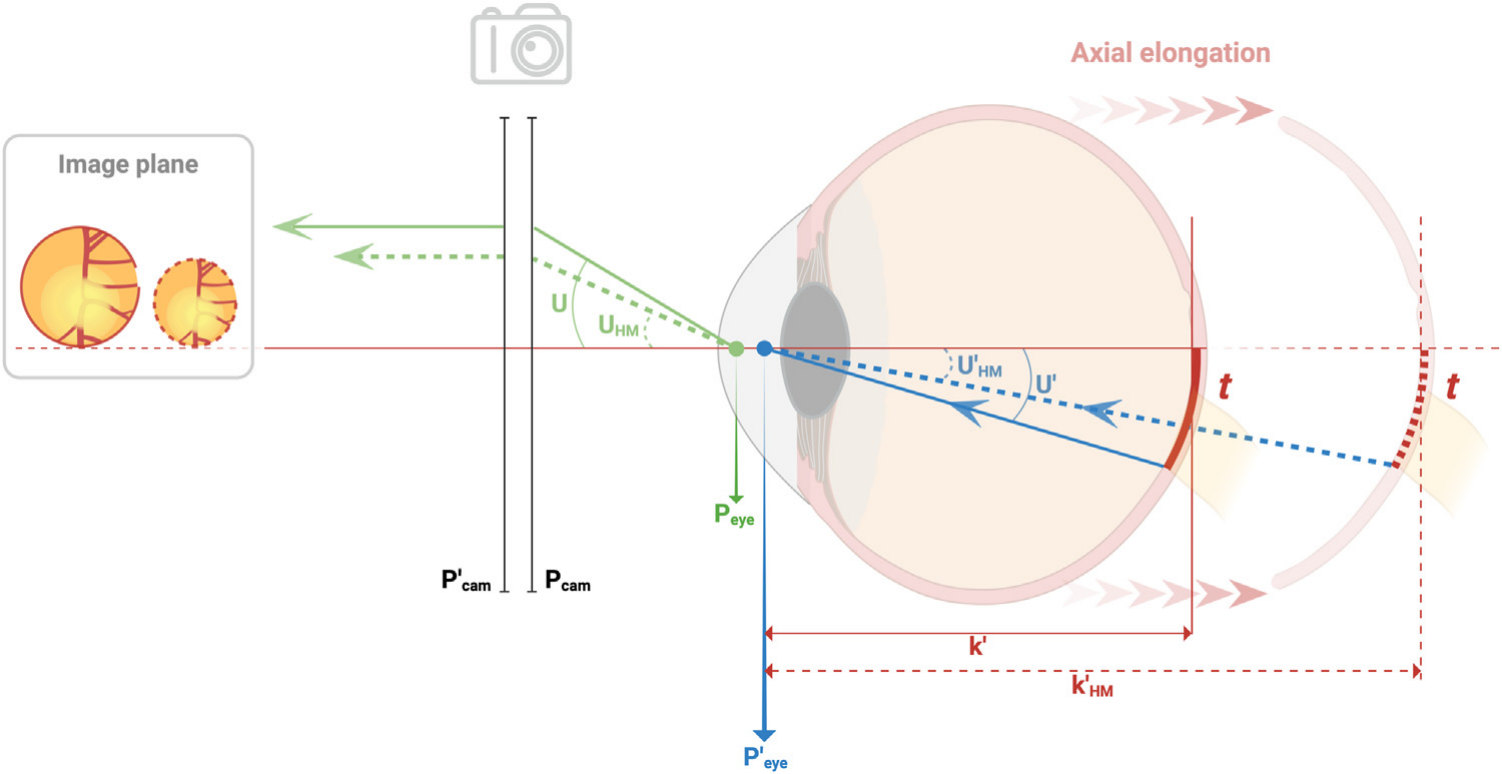
For a retinal feature imaged via fundus photography, its observed size (in the final image plane) varies—even when the true size, *t*, remains constant—following changes in the optical dimensions of the eye. The ratio of the observed size to t is the amount of transverse ocular magnification. The schematic diagram shows how myopic axial elongation leads to a decrease in ocular magnification, reducing the observed size of a retinal feature (region surrounding the optic nerve head, represented by the red curve on the retina) while its true size remains unchanged. Before axial elongation, the light ray (blue solid line with a left-pointing arrow) that gets reflected from the inferior limit of the retinal feature subtends the second principal point of the eye (blue dot denoted by *P’*_*eye*_ between the corneal apex and lens) at an angle represented by *U’*. Upon refraction by the eye’s optics, the ray emerges and subtends the first principal point of the eye (green dot denoted by *P*_*eye*_ just anterior to *P’*_*eye*_) at an angle denoted by *U*. Note that the incident angle, *U’*, is proportional to the refracted angle, *U* (i.e., one increases with the other), such that *U’* = *U*/1.336, where 1.336 is the refractive index of aqueous and vitreous humor. From here, the ray (solid green line) emerging from the eye at *P*_*eye*_ would be refracted at the anterior principal point of the camera’s optical system (P_cam_) and—assuming that the fundus camera has a telecentric design (for illustration purposes) in that its anterior focal point is made to coincide with *P*_*eye*_—the refracted ray emerging from *P’*_*cam*_ would always run parallel to the optical axis (red horizontal line passing through the corneal apex), forming an image (larger of the 2 images) in the final image plane. When the same eye becomes axially elongated (all rays are now represented by dotted lines and the subscript “HM”, for high myopia, is appended to pertinent notations), the distance between the eye’s second principal point (*P’*_*eye*_) and the retina increases from *k’* to *k’*_*HM*_, causing the angle of the ray reflecting from the same retinal feature to subtend a smaller angle, *U’*_*HM*_, at *P’*_*eye*_, which in turn decreases the refracted angle, *U*_*HM*_, ultimately giving rise to a smaller image.

In what follows, readers may find the schematic diagram in Figure 1 helpful. The camera factor, *p*, relates the angle of the ray emerging from the eye (*U or U*_*HM*_) to the size of the final image formed on the camera film (*s*), that is, *p = U* (or *U*_*HM*_*)*/*s*. Assuming telecentricity (explained in the figure), *p* may only vary between camera models and is constant for all eyes imaged with the same model. However, as the Topcon model used in this study is not telecentric (personal communication with Topcon UK), *p* may not be assumed to be constant but vary as a function of ametropia. In an earlier study, Rudnicka et al^23^ derived 10 different linear equations to estimate *p* from ametropia for 10 different nontelecentric fundus cameras. As pertinent information on *p* for the Topcon model used in the present study is proprietary and not readily available from any existing sources, we used the average of these linear equations to estimate *p*:

[2] p=0.015×SER+1.521

Created with BioRender.com.

Variations in magnification across AL are primarily attributable to the ocular factor, *q*, which is a factor that depends entirely on the vergence, K’ (in D), of the internal axis of the eye:

[3] q=17.455/K’

The vergence, K’, is inversely related to k’, which is the distance (in m) between the second principal point of the eye and the retina:

[4] K’=1.336/k’

A *smaller* vergence (K’)—alternatively a *larger* distance k’—as in the case of an axially elongated myopic eye, would result in a larger *q*, which would in turn give rise to decreasing magnification (i.e., for a given *t*, *s* decreases as *q* increases per the first equation). Put differently, as Figure 1 illustrates, this effect of decreasing magnification in axial myopia arises because an increase in distance *k’* (from *k’* to *k’*_*HM*_) following axial elongation would cause the ray that emanates from the retinal feature of interest to subtend a *smaller* angle (angle decreases from U’ to U’_HM_) at the second principal point of the eye (P’_eye_); thus, on refraction by the eye’s optics, the emergent ray (from P_eye_) that ultimately forms the image on the camera film would also subtend a *smaller* angle (angle decreases from U to U_HM_), which translates into a *smaller* image size.

Magnification can be directly derived if the value of distance *k’* is known. However, as *k’* is not readily available in practice, all existing magnification correction formulas make assumptions about the eye—to a greater or lesser degree—to approximate *k’* in order to derive *q*. These formulas can be categorized into different groups depending on which ocular parameter(s) they rely on to estimate *k’*: AL only (Bennett’s formula),^17^ SER only (Bengtsson’s formula)^12^ and CR combined with SER (Littmann’s formula):^11^

[5] Bennett’sformula:t=p×0.01306×(AL−1.82)×s

[6] $$Bengtsson’s\;formula:t=\frac{\left(1-0.017\times \text{SER}\right)}{p\times 60\;}\times s$$

[7] $$Littmann’s\;formula:t=p\times \frac{a\times {SER}^{2}-b\times \text{SER}+c\;}{100}\times s,\text{where}\;a=0.01+0.00236\times \left(\text{CR}-8\right);b=0.6126+0.0968\times \left(\text{CR}-8\right);\;\text{and}\;c=30.52+2.57\times \left(\text{CR}-8\right)$$

As discussed earlier, *p* is a constant if the imaging system is telecentric and, as is the case in the present study, varies as a function of ametropia according to equation [2] if it is not telecentric.

### Statistical Analysis

The association of AL (independent variable) with CRAE and CRVE was examined using ordinary least squares linear regression—both before and after magnification correction using Bennett’s, Bengtsson’s, and Littmann’s formulae. All models included age, sex, and ethnicity as covariates. The *lm* package in R version 4.2.2 (R Core Team 2022) was used to fit the models. Reproducible source code and tabular data directly supporting the findings of this work are freely available at github.com/fyii200/vesselCalibreAL.

## Results

Of the 82 participants included in the present study, 64 were female and 18 were male. South Asian (n = 41) and White (n = 38) were the largest self-reported ethnicities in this dataset (96%), followed by East Asian (n = 2) and Black (n = 1). The mean ± standard deviation (range) for age, SER, AL, CRAE, and CRVE (not corrected for magnification) of this dataset were 18.8 ± 2.1 (16–31) years, −0.79 ± 2.41 (−7.25 to +6.25) D, 23.82 ± 1.12 (20.65–27.33) mm, 27.4 ± 2.0 (20.8–32.4) pixels, and 35.2 ± 3.4 (27.6–47.1) pixels, respectively.

Table 1 presents the regression results when CRAE and CRVE were not corrected for ocular magnification, while Table 2 shows the corresponding results after magnification correction using different formulae. In the absence of magnification correction, there was a significant negative association between AL and vessel caliber, where every 1 mm increase in AL was associated with 0.49-pixel and 0.91-pixel reduction in CRAE and CRVE, respectively. However, after magnification correction using Bennett’s formula (“AL only” method), no significant association between AL and CRAE or CRVE was found. Similarly, after applying either Bengtsson’s or Littmann’s formula, which relied on refractive error or keratometry plus refractive error to estimate ocular magnification, the initial observation of central retinal arteriolar and venular narrowing disappeared.

**Table 1:** Association between AL (Independent Variable, bolded) and Retinal Vessel Caliber (Central Retinal Arteriolar or Venular Equivalent) without Magnification Correction

	Central Retinal Arteriolar Equivalent			Central Retinal Venular Equivalent		
	β	95% CI	*P*	β	95% CI	*P*
AL (per mm)	−0.**49**	−0.**89** to −0.**09**	**0.02**	−**0.91**	−1.**62** to −0.**20**	0.01
White	−0.**23**	−1.**09** to 0.**62**	0.**59**	−0.**20**	−**1.73** to 1.**32**	0.**79**
Age (per yr)	−0.1**4**	−0.**36** to 0.0**8**	0.2**0**	0.**14**	−0.**25** to 0.**53**	0.**48**
Male	−0.**45**	−1.**47** to 0.5**7**	0.**38**	0.**16**	−1.**66** to **1.98**	0.**86**

AL = axial length; β = unstandardized beta coefficient; CI = confidence interval; White = White ethnicity vs. non-White (South Asian, East Asian, and Black) ethnicity.

**Table 2:** Association of AL (Independent Variable, bolded) with CRAE (Top) and CRVE (Bottom) After Magnification Correction Using Formula that Relies on AL Only (Bennett’s Formula), Refractive Error Only (Bengtsson’s Formula), or Refractive Error in Combination with Corneal Radius of Curvature (Littmann’s Formula)

	Bennett’s formula		Bengtsson’s formula		Littmann’s formula	
	β [95% CI]	*P*	β [95% CI]	*P*	β [95% CI]	*P*
CRAE model						
AL	0.**13** [−0.**04** to 0.**30**]	0.**12**	0.01 [0.003–0.01]	<0.**01**	0.**06** [–0.**17** to 0.**29**]	0.**59**
White	−0.**07** [−0.**43** to 0.**30**]	0.**72**	−0.01 [−0.02 to 0.**01**]	0.**38**	−0.**18** [−0.**68** to 0.**32**]	0.**48**
Age	−0.**09** [−0.**18** to 0.0**05**]	0.**06**	−0.001 [−0.004 to 0.002]	0.**62**	−0.**41** [−0.54 to −0.**29**]	<0.001
Male	0.**03** [−0.**47** to 0.**40**]	0.**88**	−0.**02** [−0.03 to −0.00**0**2]	0.**05**	0.**20** [−0.**39** to 0.**80**]	0.**50**
CRVE model						
AL	0.**06** [−0.**24** to 0.**36**]	0.**6**9	0.01 [−0.**001** to 0.02]	0.**08**	−0.**04** [−0.**45** to 0.**37**]	0.**85**
White	−0.**03** [−0.**68** to 0.**62**]	0.**92**	−0.01 [−0.03 to 0.01]	0.**57**	−0.**18** [−1.**06** to 0.**69**]	0.**68**
Age	0.**03** [−0.**14** to 0.**19**]	0.**75**	0.00**3** [−0.00**3** to 0.01]	0.**32**	−0.**36** [−0.58 to −0.**14**]	<0.01
Male	0.**27** [−0.**50** to 1.**05**]	0.**48**	−0.01 [−0.04 to 0.01]	0.**33**	0.**57** [−0.**47** to 1.**62**]	0.**28**

AL = axial length; β = unstandardized beta coefficient; CI = confidence interval; CRAE = central retinal arteriolar equivalent; CRVE = central retinal venular equivalent; White = White ethnicity vs. non-White (South Asian, East Asian, and Black) ethnicity.

We additionally examined the impact of inappropriately treating the Topcon fundus camera used in this study as telecentric, that is, by assuming the camera factor *p* in equation [1] to be a constant. Using this inappropriate assumption, we observed a bias toward increasing CRAE (Bennett’s formula β: 0.30, *P* < 0.001; Littmann’s formula β: 0.25, *P* = 0.03; Bengtsson’s formula β: 0.004, *P* = 0.22) and CRVE (Bennett’s formula β: 0.29, *P* = 0.04; Littmann’s formula β: 0.22, *P* = 0.26; Bengtsson’s formula β: 0.002, *P* = 0.76) with increasing AL, where the beta coefficients were positive or became more positive.

## Discussion

In the absence of magnification correction, retinal vessel caliber decreases as AL increases. However, this association merely reflects the optical effect of reduced magnification in longer eyes, as arteriolar and venular narrowing is no longer evident after correcting for ocular magnification, regardless of the correction formula applied. Additionally, using a constant camera factor, *p* (an inappropriate assumption), for a nontelecentric imaging system biases the association toward arteriolar/venular widening in longer eyes.

Findings from 2 earlier studies also lend credence to an optical origin of arteriolar and venular narrowing in myopia. In predominantly ethnic Chinese children aged 7 to 9 years from the Singapore Cohort of Risk Factors for Myopia, increasing AL was associated with decreasing CRAE and CRVE,^9^ but these associations disappeared (*P* > 0.05) after the analysis was repeated after magnification correction using Bengtsson’s formula. Likewise, in white adults aged ≥49 years from the Blue Mountains Eye Study, the initially significant association between myopic refraction and smaller CRAE or CRVE became nonsignificant after Bengtsson’s formula was applied.^10^ Having said that, findings from Li et al,^7^ which looked at Singapore-based Chinese toddlers aged 1 to 3 years, and Lim et al,^8^ which focused on Malay adults with and without diabetes aged 40 to 80 years from the Singapore Malay Eye Study, weaken our argument for an optical origin of myopic reduction in CRAE/CRVE, as both studies still found evidence of narrower central retinal arterioles and venules in eyes with greater AL after correcting for ocular magnification using Bengtsson’s formula. These discrepancies, however, may be explicable by the reasons discussed below.

First, in toddlers where anterior ocular parameters, particularly the radii of curvature, refractive index, and thickness of the crystalline lens, are still undergoing active and rapid changes,^24^ the use of Bengtsson’s formula in the former study^7^ was not appropriate, given that the formula (or any other formula examined herein) assumes a constant, “normal” value of around 20 D for the equivalent power of the lens.^13^ Compared with adults or older children, these assumptions are far from correct in toddlers aged 1 to 3 years where the lens power is around 32 to 37 D on average, with the greatest temporal changes also occurring during this period.^24^ The reported magnification correction factor (1−0.0017×SER) also appears inconsistent with the original Bengtsson’s derivation (1−0.017×SER). While this was quite possibly a typographical error, magnification correction using the former correction factor would erroneously bias the observed association toward arteriolar and venular narrowing in myopia, and vice versa in hyperopia. To illustrate, using the former correction factor, the calculated *q* in equation [1] would be *underestimated* in a myopic eye with an SER of −10 D (i.e., 1.02 vs. the correct value, 1.17)—causing the derived true vessel caliber, *t*, to be underestimated—but *overestimated* in a hyperopic eye of +10 D (i.e., 0.98 vs. 0.83)—causing *t* to be overestimated.

**Figure 2:**
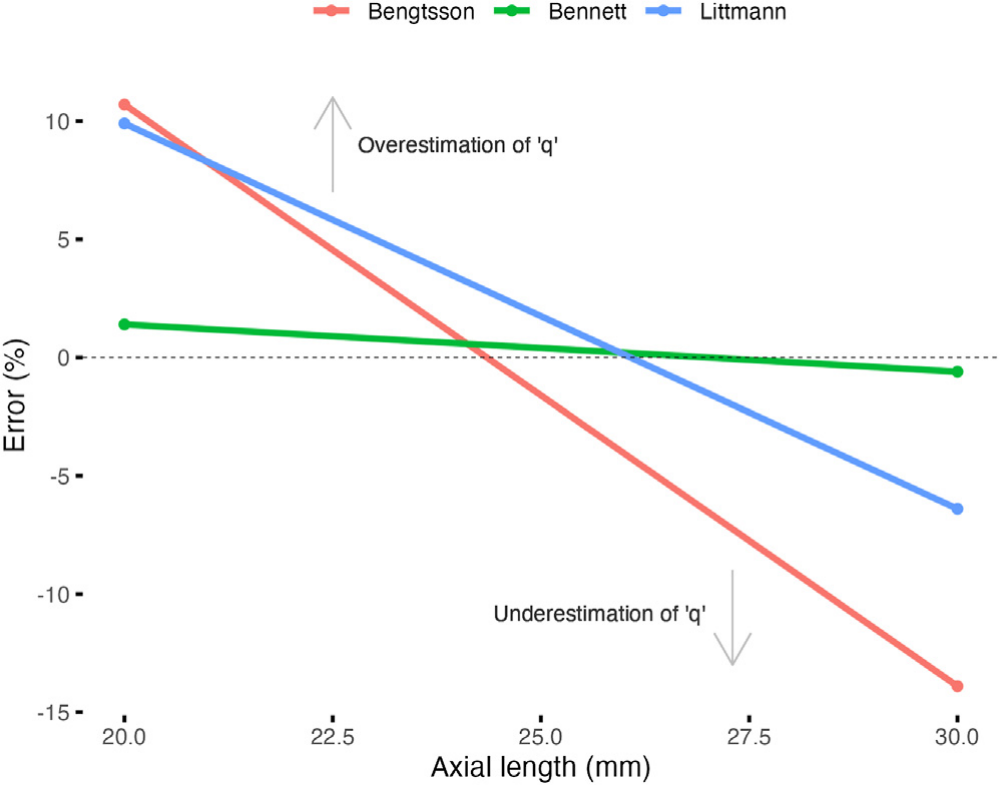
Error in estimating the ocular factor *q* using different magnification correction formulae as a function of axial length. A positive (negative) error indicates that the ocular factor *q* is overestimated (underestimated), which in turn indicates that the magnification-corrected central retinal arteriolar or venular equivalent is overestimated (underestimated). All formulae exhibit proportional bias, to a lesser or greater extent, in that *q* is overestimated in shorter eyes and underestimated in longer eyes, with Bengtsson’s formula having the highest bias (slope: −2.46), followed by Littmann’s formula (−1.63) and Bennet’s formula (−0.2; virtually free of the bias).

In an experiment conducted to compare the accuracy of different magnification correction formulae, Garway-Heath et al^13^ measured the “error” of each formula by calculating the deviation of the ocular factor *q* obtained with each formula from that obtained with a “gold-standard” formula that relied on the greatest number of ocular parameters (i.e., AL, refractive error, keratometry, anterior chamber depth, and crystalline lens thickness). It was concluded that Bennett’s formula, which relied on AL, was the most accurate due to the close relation between AL and k’ (Fig 1) and, as can be seen in Figure 2, its magnitude of error varied very little across a wide range of AL (i.e., very small proportional bias). While it is clear that all formulas overestimated *q* in shorter eyes and underestimated it in longer eyes, to a lesser or greater extent, Bengtsson’s formula exhibited the highest proportional bias (i.e., steepest slope in Fig 2). Since the calculated true size of a retinal feature, *t* in equation [1], is proportional to the ocular factor *q,* an underestimation of *q* in longer eyes would cause the derived/calculated *t* to be underestimated, and vice versa for the overestimation of *q* in shorter eyes. In other words, a high proportional bias would cause the magnification-corrected CRAE or CRVE to be erroneously too small in longer eyes and too big in shorter eyes, which may also partly explain why myopic vascular narrowing was still noted in some studies cited in the previous paragraph after applying Bengtsson’s formula.

Note that the data were extracted from Figure 1 in Garway-Heath et al^13^ using WebPlotDigitizer at https://automeris.io/v4/ and subsequently replotted.

As noted in our previous work, magnification correction using Littmann’s formula resulted in arteriolar and venular widening as refractive error increased in the myopic direction.^2^ However, in the present study, the use of Littmann’s formula did not produce similar results with increasing AL while correctly assuming the camera to be nontelecentric. Given that camera models manufactured by the same company tend to have similar designs (e.g., Zeiss cameras are typically telecentric, while Canon cameras are usually nontelecentric),^23^ we assumed in our previous work that the fundus camera integrated within the Topcon 3D OCT 1000 used in the UK Biobank was of telecentric design because another Topcon model (TRC-50DX) was previously reported to be telecentric.^25^ Besides, another study using a Topcon 3D OCT 2000 device, closely related to the model used in our previous work, also assumed the imaging system to be telecentric.^26^ However, we recently learned that this assumption may not hold for Topcon fundus cameras.^27^ Thus, the discrepancy in results between our previous and current studies may be due to the use of a constant camera factor *p* in our earlier work—considering that when making the inappropriate assumption that *p* was constant in this work, we observed a bias towards vascular widening with increasing AL. Indeed, after repeating the analysis from our previous work^2^ using the same magnification correction approach as that applied herein (i.e., varying *p* as a function of ametropia), arteriolar and venular widening was no longer evident with more negative SER. Findings for other dimensional measures, including optic disc area and optic disc-fovea distance, remained unaffected.

A strength of this study is the use of healthy young adults, whose ages are old enough for the ocular assumptions of the magnification correction formulae to be reasonably valid (e.g., equivalent power of cornea and lens close to adult “normal” values) but also young enough to minimize the influence of age-related systemic changes, such as blood pressure. Additionally, the adoption of Bennett’s formula for magnification correction enabled us to draw stronger conclusions compared with studies applying other, less accurate formulae that are more susceptible to proportional bias (Fig 2). However, a limitation of the formulae used in this work (as in all existing studies), which was discussed in our previous work,^2^ is their reliance on the imperfect assumptions that all ray angles are small (paraxial approximation) and that the eye is rotationally symmetric. The use of a relatively small sample, drawn from a university-based population, may also limit the external validity of our study. Furthermore, as information pertaining to the telecentricity of newer fundus imaging systems is not available, particularly those manufactured by Topcon, the equation for deriving the camera factor, *p*, had to be inferred. Future studies using telecentric fundus cameras or high-resolution adaptive optics imaging systems could help further validate our findings. Finally, it is important to note that imaging features do not necessarily reflect ocular physiology. Thus, while we found no evidence of a biological reduction in vessel caliber in axially elongated eyes, this does not provide direct evidence that ocular blood flow remains unchanged in myopia.

In conclusion, axial elongation results in apparent arteriolar and venular narrowing due to reduced ocular magnification. Proper magnification correction eliminates this observed reduction in vessel caliber, while incorrect assumptions about camera design (telecentricity) during magnification correction lead to apparent vascular widening. Our findings, therefore, suggest an optical rather than biological link between myopia and vessel caliber. The optical influence of ocular biometry and camera design on dimensional measures is a crucial, yet often overlooked, methodological consideration in oculomics research investigating retinal biomarkers of systemic conditions.^28^ For example, studies exploring the association between retinal vessel caliber and cognitive decline or dementia often overlook the importance of magnification correction, making it difficult to determine if the reported associations—whether significant or not—are confounded by magnification variations caused by differences in AL between groups,^29^, ^30^, ^31^, ^32^ especially given the previously noted link between myopia and cognitive decline.^33^^,^^34^ Future oculomics studies are recommended to consider magnification correction and verify whether telecentric (constant *p*) or nontelecentric (varying *p*) correction is appropriate.
